# Assessing realism of artificial intelligence-generated colorectal polyp images: International multicenter blinded reader study

**DOI:** 10.1055/a-2865-3480

**Published:** 2026-05-15

**Authors:** Philipp Sodmann, Ronja Weber, Valentin Wettstein, Ioannis Kafetzis, Yun Chiang, Venkatesh Parayitam, Michela Pauletti, Konstantinos Kouladouros, Alexander Meining, Alexander Hann

**Affiliations:** 1 Interventional and Experimental Endoscopy (InExEn), Department of Internal Medicine 2University Hospital WürzburgWürzburgGermany; 2 Bavarian Cancer Research Center (BZKF)WürzburgGermany; 3 Central Interdisciplinary Endoscopy, Department of Hepatology and Gastroenterology 14903 Charité - Universitätsmedizin BerlinBerlinGermany

**Keywords:** Endoscopy Lower GI Tract, Polyps / adenomas / ..., Generative AI, Training, Image and data processing, documentation

## Abstract

**Background and study aims:**

Prevention of colorectal cancer relies on detection and characterization of polyps during colonoscopy, yet access to large, shareable training datasets is limited. To address this issue, we developed a diffusion-based artificial intelligence (AI) model to generate synthetic polyp images in high-resolution and assessed their perceived realism in an international blinded reader study.

**Patients and methods:**

Fifty-three endoscopists from 46 centers across 14 countries evaluated 20 real and 20 AI-generated images in random order using our web platform Lutetia. Experts classified the images as either real or synthetic and rated their confidence. The primary endpoint was sensitivity of identifying synthetic images. Secondary endpoints were recognition of real images and overall accuracy. The trial was registered at clinicaltrials.gov with the identifier NCT07108569.

**Results:**

Sensitivity for detecting AI-generated images was 66% (95% confidence interval [CI] 65%-67%) with a specificity of 80% (95% CI 79%-81%); overall accuracy was 73% (95% CI 72%-73%). Low-confidence decisions were more frequent for AI-generated images and associated with longer annotation time ( *P* < 0.001).

**Conclusions:**

Difficulties in differentiating synthetic from real images together with lower confidence and longer decision times shows that our diffusion model generates highly realistic polyp images.

## Introduction

Colorectal cancer (CRC) is the third most common malignancy and the second leading cause of cancer-related death worldwide. Colonoscopy is central to CRC prevention, therefore, training of clinicians in identification and classification of polyps is essential 1 . Beyond exposure in clinical routine, additional training should be performed using selected endoscopic images of high quality and established ground truth 2 . However, concerns are raised regarding protection of individual privacy when patient data are used outside of direct patient care 3 . Consequently, the process of data sharing is lengthy and involves obtaining ethical approval, gaining informed patient consent, and anonymizing data. All these steps require considerable time and resources 4 . As a result, availability of publicly accessible datasets remains limited. In addition, collections of large and diverse datasets are essential for research in the medical field. This has become even more relevant with the advent of artificial intelligence (AI). For endoscopy, AI-based systems have been developed to assist physicians with polyp detection 5 6 , characterization 7 8 , and size measurement 9 . Development of reliable AI systems also requires large high-quality datasets, covering a diversity in devices, centers, and pathologies to generalize.

Synthetic medical images, which are independent of sensitive patient data, have the potential to provide a welcome alternative. Recent advancements in the field of AI do allow for generation of high-quality images through generative models (GMs) 10 . AI-based image generation has been the focus of research across different medical specialties. Augmentation of datasets through synthetic images for development of AI-based detection systems, in particular, has been of great interest 11 . GMs have been applied across different modalities, such as radiology 12 and pathology 13 .

Several previous works have used GMs to create synthetic endoscopic polyp images 14 15 16 . These studies, however, were generally limited by a small number of experts, single-center reader groups, and images generated in relatively low resolution and often mixing different generation methods for synthetic images of very different quality in the same study. However, to date, no studies have evaluated the realism of these images from a human perspective through an international reader study including a high number of experienced endoscopists.

Based on over 40 million endoscopy frames from over 7,000 examinations, we developed and trained a latent diffusion model (LDM) 17 that generates high-resolution images of colon polyps. To assess the realism of these images, we conducted an international multicenter blinded reader study.

## Materials and methods

### Data selection and AI generation

For AI training, over 7,000 colonoscopy high-resolution videos with over 40 million single image frames from eight centers were retrospectively identified. Using a pretrained multi-label AI model as previously described 18 helped to describe images semantically with predefined classes including visibility of polyps, overall image quality, and presence of resection instruments.

A LDM was built in Python (Supplementary data). During image generation, the model was set to generate high-quality images without visible instruments, blood, or resection wounds.

Real images were selected from image reports to display high-quality polyp images. Synthetic images were generated with a conditioning vector that used the multi-label AI predictions of real images containing polyps. The generated images were manually reviewed and only kept if they contained polyps. Because the model does not guarantee a polyp to be present in every generated image and the lesion subtype could not be controlled, the images were manually reviewed and excluded if the image did not contain a visible polyp. From this curated pool, 20 images were randomly selected.

### Study design

We conducted an international, multicenter blinded reader study to evaluate the perceived realism of the generated high-resolution polyp images, inviting 69 endoscopists from 15 countries to participate in the study. Inclusion criteria required active endoscopic practice with at least 400 colonoscopies performed. For subgroup analysis, experts were defined with experience performing more than 1000 colonoscopies.

Annotation was performed using Lutetia, an interactive, web-based annotation and teaching tool developed specifically for this study. Lutetia allows users to view and annotate polyp images in a standardized interface, eliminating need for manual file copying or spreadsheet completion, while also obfuscating information such as file names to eliminate bias ( **Supplementary Fig. 1** ).

**Fig. 1:**
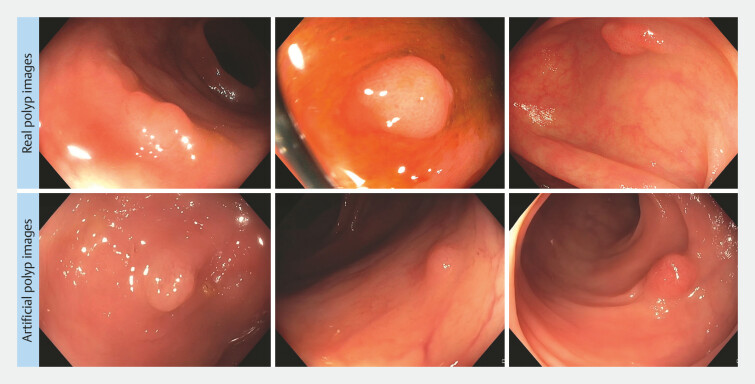
Examples of real (upper row) and AI-generated polyp (lower row) images.

Participants were presented with a series of 40 endoscopic images in random order and were asked to classify each image as to whether it was real or AI-generated, along with rating their confidence as either high or low for each decision. The dataset contained 20 real as well as 20 AI-generated polyps. Real and synthetic images contained examples of four different Paris classes: Ip, Is, IIa, and IIb. The participants were blinded regarding the quantity of real and AI-generated images. In addition, participants had the option to provide feedback on their experience after completing the survey. Examples of real and AI-generated polyps are displayed in Fig. 1 .

Primary endpoint was the sensitivity of identifying artificial polyp images as AI-generated, secondary endpoints included the ability to identify real images as real as well as the overall classification accuracy. Furthermore, we analyzed the self-rated confidence and duration of each individual annotation.

### Embedding space analysis

DINOv2 19 embeddings were computed for the training data as well as for the real and synthetic study images. Cosine similarity was used to identify nearest neighbors and assess image memorization from the training data. Fréchet Distance (FD) in the DINOv2 embedding space was calculated to compare distributional overlap between the three sets of images. A UMAP 20 projection was used to visualize the embedding distribution in a two-dimensional manifold.

### Statistical analysis

Statistical analyses were conducted using Python 3.11. Annotation durations between experience groups were compared using the non-parametric Mann-Whitney U test. Differences in annotation duration across confidence levels were assessed using a one-way ANOVA and confirmed with the non-parametric Kruskal-Wallis test. Associations between categorical variables were examined using chi-square tests of independence. Performance metrics (accuracy, specificity, and sensitivity) between experience groups were compared using the Mann-Whitney U test, with 95% confidence intervals (CIs) estimated via bootstrap resampling (10,000 iterations).

### Ethical considerations

The study was approved by the local medical ethics committee at the Julius Maximilian University Würzburg (No. 2022120701). All procedures were in accordance with the Helsinki Declaration of 1964 and later versions.

## Results

### Participant characteristics

Of 69 invited physicians, 56 participated in the study. Three participants had to be excluded due to not completing the study tasks. This resulted in inclusion of 53 participants from 46 centers and 14 countries ( **Supplementary data** ). Forty-six of the participants were expert endoscopists who had performed over 1000 colonoscopies, whereas the remaining seven had performed between 400 and 1000 colonoscopies.

### Reader performance and confidence levels

**Fig. 2:**
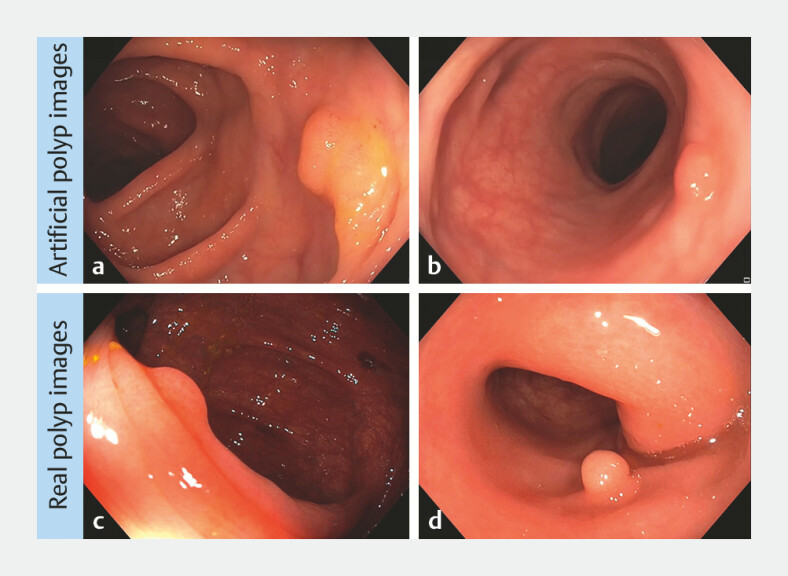
Images representing extremes of image classification by experts. The upper row displays AI-generated images and the bottom row real images. According to participant annotations, **a** is the AI-generated image most often classified as real, **b** is the AI generated example most often classified as synthetic, **c** is the real image that is most frequently classified as real, and **d** shows the real image most common misclassified as synthetic.

Participants in the study were able to detect AI-generated polyps with a sensitivity of 66% (95% CI 65%-67%) and a specificity of 80% (95% CI 79%-81%). Because participants were shown an equal number of real and AI-generated polyps, the corresponding sensitivity and specificity for the classification of real images were 80% (95% CI 79%-81%) and 66% (95% CI 65%-67%), respectively. Overall classification accuracy of images was 73% (95% CI 72%-73%). Fig. 2 shows examples of the most frequently correctly and incorrectly annotated images.

**Fig. 3:**
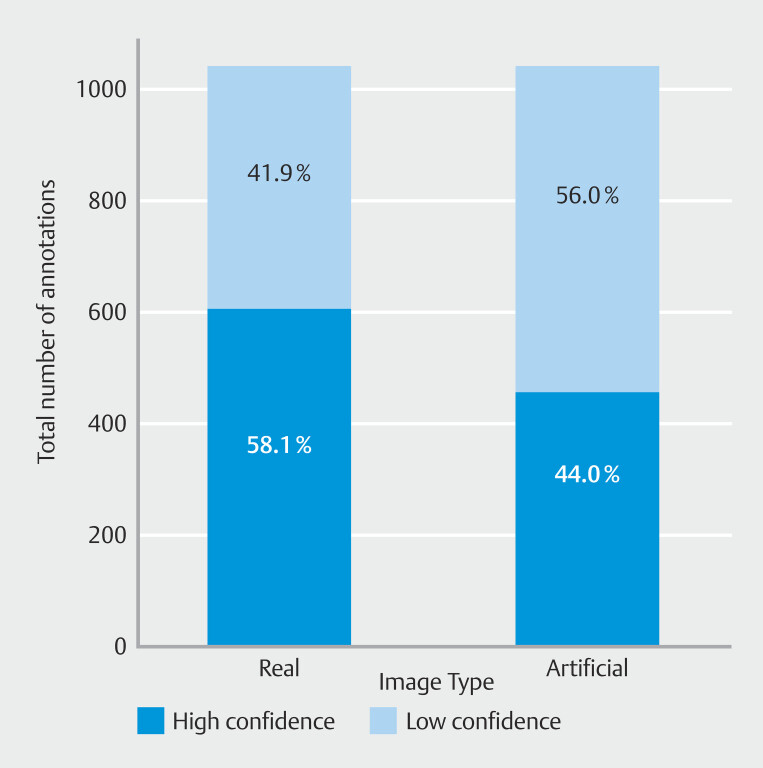
User confidence when shown real and synthetic images. The reported confidence was lower when annotating AI generated images.

Assessment of AI-generated polyps was associated with significantly increased uncertainty ( *P* < 0.001). Expert endoscopists with more than 1000 colonoscopies reported a high confidence in their decision in 52% of cases, compared with 48% in the less experienced group. Classifying images with low confidence was associated with significantly longer annotation time ( *P* < 0.001). Participant confidence during classification of real and AI-generated polyps is displayed in Fig. 3 .

There was no significant difference in sensitivity for real polyps regarding experience level. However, less experienced participants had significantly higher accuracy ( *P* < 0.001) and specificity ( *P* < 0.001) compared with experts ( **Supplementary Fig. 2** ).

Unfortunately, we did not collect structured feedback for each image to identify reasons for the annotations. Still, participants were able to send us qualitative free text feedback after completion of the study. Examples of comments are provided in the **Supplementary Data** .

### Embedding space analysis

Visual inspection of the closest training images revealed that, although the synthetic study images shared a similar overall endoscopic scene structure, they were distinct from the frames used as training data ( **Supplementary Fig. 3** ). The UMAP projection showed that the synthetic and real study images were evenly distributed within the region of polyp images in the training data ( **Supplementary Fig. 4** ).

Synthetic images showed a mean nearest-neighbor cosine similarity to the training set of 0.893 (standard deviation [SD] 0.022, range 0.840–0.923), compared with 0.873 (SD 0.040, range 0.840–0.925) for real images. The FD between the synthetic and real images shown in the study (0.406) was lower than both the FD between training and shown real images (0.642) and the FD between synthetic and training images (0.698).

## Discussion

In this study, participants identified real polyps with a relatively high sensitivity of 80%, which indicates a strong understanding of visual characteristics of real polyps. However, in many cases, AI-generated polyps were incorrectly classified as real, which is reflected by the low specificity of 66%. These findings, together with significantly higher uncertainty of the participants when presented with an AI-generated image, underscore the ability of our AI model to create images perceived as realistic. These results match the qualitative feedback we received from the participants. Several participants noted that they were mostly guessing because the images were difficult to differentiate visually and they had to rely on minimal cues such as mucosal reflections and overall blurriness. According to signal detection theory, when an observer is required to make a forced decision between two image classes, performance on the decision depends on perceivable differences between the classes. A perfect image generator would produce synthetic polyps that do not contain any signal to support that choice and, therefore, are indistinguishable from real images 21 . This would lead to reader performance close to random chance with about 50% accuracy in a balanced dataset. Over all the images, readers correctly identified the origin of the image in 73% of presented examples, which corresponds to a chance-corrected accuracy of 46% in a balanced forced-choice task. These results suggest that the generated images are sufficiently realistic in their visual features (polyp shape, surface patterns, mucosa, and vessels) to be rated as real.

Importantly, the perceived realism does not appear to be a result of memorization because the synthetic images are visually distinct from their nearest neighbors in the training data. Slightly higher similarity of synthetic images to training data is expected because the model was trained on this dataset. Furthermore, the low FD between the synthetic and the real study images indicates that both groups share a similar distribution, which indicates that they were both selected by similar criteria.

Liu et al. also conducted a reader study to evaluate realism of polyp images generated by their diffusion-based model “Polyp-Gen” and two other GMs 22 . In the study, participants were presented with a total of 400 polyp images, 100 real and 100 generated by each model. The two participants misclassified polyps generated by “Polyp-Gen” as real in 90% and 82% of cases, respectively. However, images generated by both other models were identified as synthetic in significantly higher percentages, indicating less realistic images. As a result, participants may have classified “Polyp-Gen” images as real not based on their similarity to real polyp images, but rather, in comparison to less realistic images from the other models. In addition, the significance of the study is limited by the small number of participants.

In a study by Yoon et al., realism of AI-generated images of sessile serrated lesions (SSLs) was evaluated by four experts 15 . The image collection consisted of 25 real images and 25 synthetic images, which is similar to our study design. Overall accuracy for the correct classification was 63% compared with 73% in our study. Sensitivity for identification of real polyps was almost identical in both studies, whereas specificity of 47% was even lower than in our study, indicating high authenticity of AI-generated SSLs. However, the synthetic polyp images used in both studies are difficult to compare because the shape, color, surface features, and general appearance of SSLs differ significantly from the typical adenomas presented in our study.

Ioanovici et al. evaluated 24 low-resolution images of 256 × 256 pixels in a single-country cohort 14 . The dataset contained eight real images, eight images after augmentation, four images generated by Cycle GAN, and four images using a diffusion-based network. In this study, all images generated by Cycle GAN were detected as synthetic whereas the diffusion-generated images were correctly identified in 22.7%.

In contrast, our study used 40 high-resolution images. All synthetic images were generated using the same diffusion model to avoid potential contrast bias, a well-known effect in psychology, in which clearly unrealistic images might influence judgement of subsequent examples.

To our knowledge, this is the first study evaluating realism of AI-generated polyp images through a reader study including an international multicenter cohort of experienced endoscopists. Because we intend to use synthetic polyp images primarily for training physicians rather than developing AI-models, their visual realism from a human perspective is of particular importance.

This study has several limitations. First, the set of synthetic polyps follows the distribution of those seen in clinical practice and thereby primarily represents common polyp morphologies. The model is not capable of reliably generating rare types such as lateral spreading tumors. Adding polyps of rare or atypical appearance will increase diversity of images generated by our model. Second, the confidence assessment provided to participants was limited to a binary choice (high or low) without the option for intermediate levels. This restriction may have oversimplified participant diagnostic confidence and limited the informative value of the confidence data. Third, we cannot rule out that features like sharpness and mucosal reflections of real images were identified by participants as distinguishable features between real and artificial images and, thus, resulted in the high sensitivity of detecting real images. Fourth, the main aim of the study was to find out whether the generated images look realistic in order to be used for medical training later. Thus, there was neither a lecture on how to differentiate real from artificially generated polyps prior to the study, nor did we evaluate if any of the participants had in-depth knowledge about features of artificially generated images. Fifth, the AI-generated images used in this study were drawn from a manually curated pool. Images not containing polyps were excluded prior to random selection. Similarly, the real images were selected from endoscopic reports rather than drawn randomly from the video frames used to train the model. However, because the generated images trained on video frames may be blurry or only show an incomplete polyp, manual curation was required to ensure comparable image quality between the real and synthetic images. Moreover, we built the generative model for a teaching platform for Paris classification in a subsequent study and our approach reflects a realistic scenario in which the model is used for teaching where the images need to undergo quality control before being shown. The primary aim of the study, therefore, was to assess whether the model can produce perceptually realistic polyp images whereas the overall success rate was not part of the study. Lastly, although we can show that all the images from the study are distinct from the training data ( **Supplementary Fig. 3** and **Supplementary Fig. 4** ), it cannot be excluded that the model is able to reproduce entire images from the training data, which is highly unlikely due to the stochastic nature of the diffusion process.

## Conclusions

In conclusion, frequent misclassification of synthetic images as real demonstrates that our diffusion model is capable of generating polyp images with high perceived realism. Even experienced endoscopists were unable to reliably distinguish AI-generated from real polyps. These synthetic polyp images provide an alternative to sensitive patient-derived data and will be used on a training platform for classification of precancerous lesions in future studies.

## Supplementary Material

Supplementary Material Zusatzmaterial
