# Supplementary material for: Assessing realism of artificial intelligence-generated colorectal polyp images: International multicenter blinded reader study
Source: Endosc Int Open. 2026 May 15;14:a28653480. doi: 10.1055/a-2865-3480 (PMC13289962; doi:10.1055/a-2865-3480)
Supplement: Supplementary file 1 — Supplementary Material [file 10-1055-a-2865-3480_28666947.pdf]

List of participating countries

Germany, Romania, UK, Belgium, Portugal, USA, Italy, Poland, Israel, Canada, Turkey, Netherlands, Norway, Czech Republic.

Diffusion model

We implemented a LDM which operates in two stages. The first stage generates a latent representation with a size of 96 x 72 pixels using a U-Net-based denoising diffusion process. In the second stage a VQ-VAE decoder performs reconstruction of the latent representation into an image with a resolution of 768 x 576 pixels.

The VQ-VAE was trained from scratch using frames from recorded endoscopies. The encoder and decoder each consist of three strided convolutional layers with 128 hidden channels and residual blocks which compress the spatial dimensionality into 1/64 of its original resolution. A codebook size of 1024 was used for quantization and updated via exponential moving averages. Training used a combination of L1 loss, multi-scale SSIM, LPIPS perceptual loss, and a commitment loss to anchor the encoder outputs to the codebook. The model was trained using AdamW with cosine annealing until the validation loss plateaued after 63 epochs.

The diffusion model was created using the diffusers library and was trained using a Denoising Diffusion Probabilistic Modelling objective with a 1000 step sigmoid noise schedule and used with 100 steps during image generation. The model was a U-Net and consisted of six down-sampling and up-sampling stages with symmetric skip-connections. Channel depth increased with each stage (128, 128, 256, 384, 512, and 768). Each stage contained two ResNet blocks and deeper layers contained spatial self-attention to improve global context modeling.

To enable conditional image synthesis, output of a multi-label classifier was used to condition the model during training. A linear layer aligned the conditioning features with model dimensionality for the following classes: appendiceal orifice, biopsy forceps, digital chromoendoscopy, ileocecal valve, ileum, low image quality, outside of the body, polyp, resection-wound, snare, and blood. This conditioning allowed limited control of the content of the generated images during inference. Due to copyright reasons, the full source code can only be shared upon request.

Lutetia annotation platform

The platform consists of two independent components: A frontend, written in Typescript, uses the Angular framework in the participants browser and allows display, interaction, and annotation of data. The backend which is written in Python uses FastAPI to handle authentication and securely

serves the images to the frontend. This web-based approach allowed participants to take part in the study without downloading data or installing software.

Because the dataset contains anonymized images from examinations, all communication was performed over an encrypted connection after authentication. This approach also prevents leakage of file extensions, which can be different for the exported real images. Furthermore, it prevents third parties from scraping and collecting any sensitive data.

The platform was hosted on a secure server at the University Hospital Würzburg. All accounts were provided to the participants and open registration was disabled. Unique authentication links were sent to each participant, which were used instead of passwords.

Qualitative feedback

Examples of comments using the free text field:

“I did the real/fake questionnaire (it was fascinating- I was mostly guessing as for the vast majority the cases looked very realistic)”

“Very exciting. The AI is really good; I often wasn't sure whether the pictures were fake or real”

“Apart from a few minimal cues (image sharpness, reflections), you don't really have a chance to tell them apart”

“Taking part in this study has already given me some trouble. In fact, I couldn't clearly distinguish between real and fake. I also wouldn't know what criteria I should use to identify “fake”?”

Supplementary Fig. 1 Screenshot of the annotation platform.

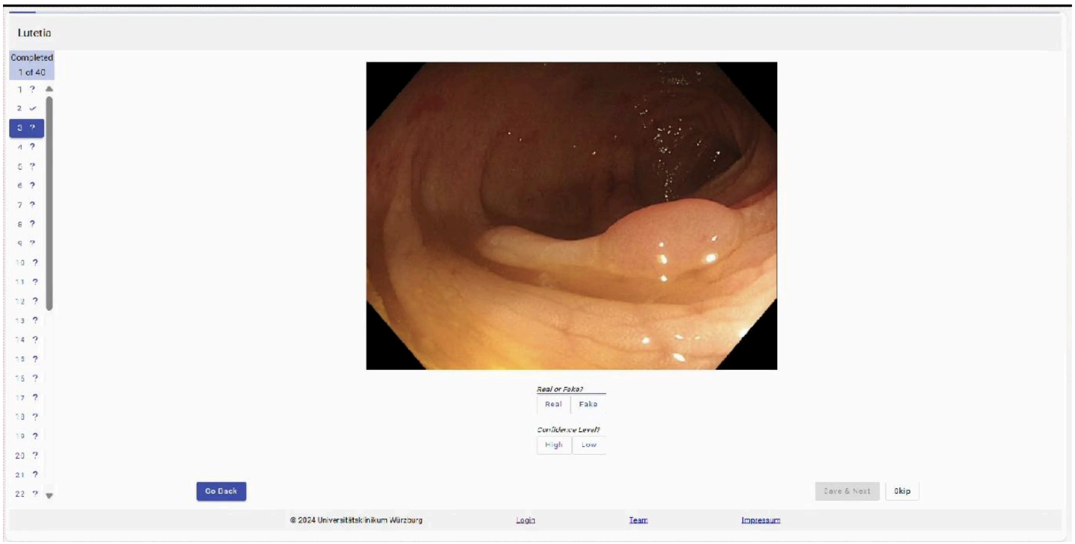

Supplementary Fig. 2 Performance comparison between more- and less-experienced endoscopists.

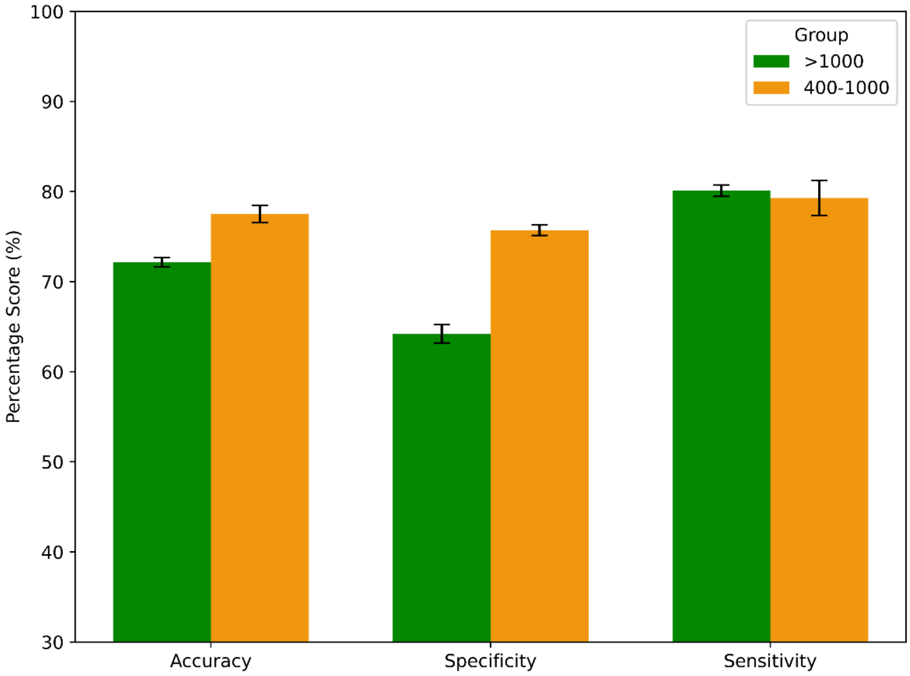

Supplementary Fig. 3 The three nearest neighbors in the training dataset from DINOv2 embeddings of each synthetic image used in the study. The generated images look similar regarding the overall structure of the image, yet distinct from the training data.

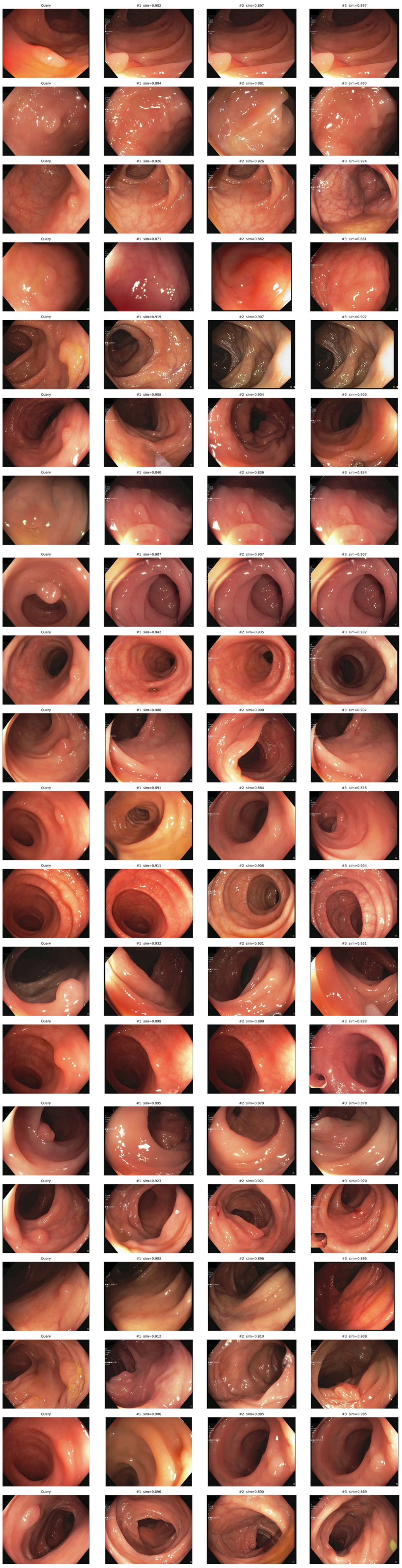

Supplementary Fig. 4 UMAP projection of DINOv2 embeddings from a random subsample of 50,000 images from the training dataset. The UMAP highlights polyp images (purple), real study images (blue x), and synthetic study images (orange Δ) overlaid. Synthetic images are distributed throughout the polyp-containing region of the training embedding space. For reference, semantic subgroups including low image quality (blue o), grasper (green o), and snare (orange o) images are highlighted, forming partially overlapping yet distinct clusters that confirm semantic interpretability of the embedding space.

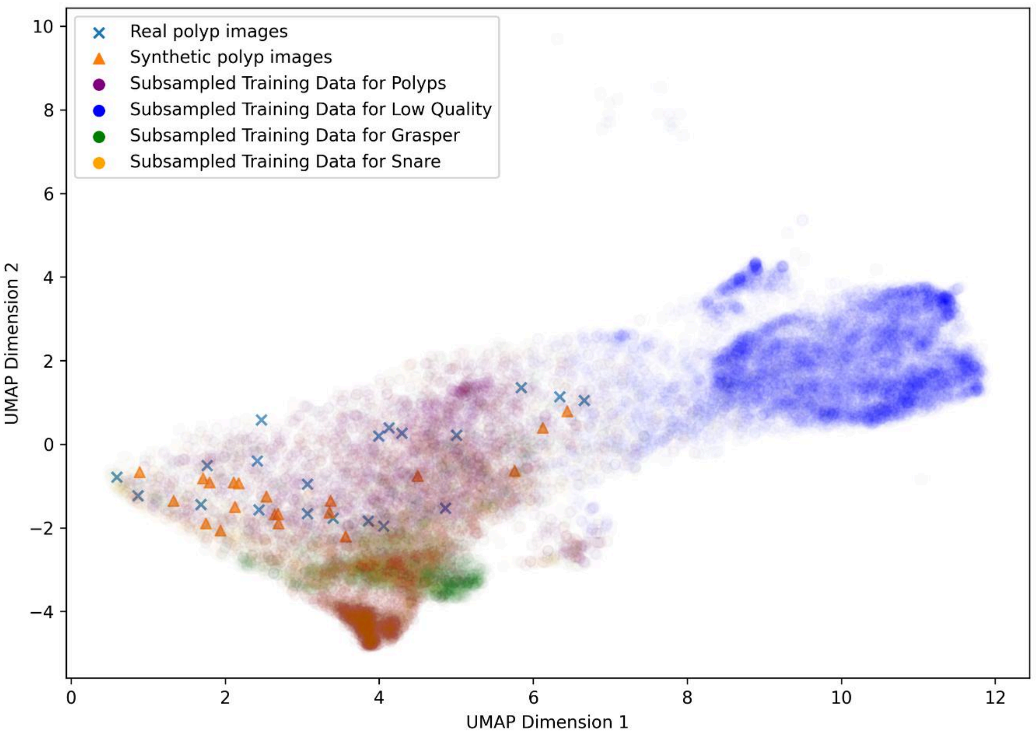

Endosc Int Open | DOI 10.1055/a-2865-3480 | © The Author(s).
